# Suppressing breast cancer by exercise: consideration to animal models and exercise protocols

**DOI:** 10.20463/pan.2020.0011

**Published:** 2020-06-30

**Authors:** Jea Jun Lee, Suji Beak, Sang Hyun Ahn, Byung Seok Moon, Jisu Kim, Kang Pa Lee

**Affiliations:** 1 Laboratory Animal Center, Osong Medical Innovation Foundation, Cheongju Republic of Korea; 2 Research and Development Center, UMUST R&D Corporation, Seoul Republic of Korea; 3 Department of Anatomy, Semyung University, Jecheon Republic of Korea; 4 Department of Nuclear Medicine, Ewha Womans University College of Medicine, Seoul Republic of Korea; 5 Physical Activity and Performance Institute, Konkuk University, Seoul Republic of Korea

**Keywords:** Breast cancer, Exercise, Breast cancer mouse models, Exercise protocols, Exercise effects

## Abstract

**[Purpose]:**

Exercise is thought to have a significant effect on chemotherapy, and previous studies have reported that exercise can increase patient survival. Thus, in this review, we aimed to summarize various animal models to analyze the effects of exercise on breast cancer.

**[Methods]:**

We summarized types of breast cancer animal models from various reports and analyzed the effects of exercise on anti-cancer factors in breast cancer animal models.

**[Results]:**

This review aimed to systematically investigate if exercise could aid in suppressing breast cancer. Our study includes (a) increase in survival rate through exercise; (b) the intensity of exercise should be consistent and increased; (c) a mechanism for inhibiting carcinogenesis through exercise; (d) effects of exercise on anti-cancer function.

**[Conclusion]:**

This review suggested the necessity of a variety of animal models for preclinical studies prior to breast cancer clinical trials. It also provides evidence to support the view that exercise plays an important role in the prevention or treatment of breast cancer by influencing anticancer factors.

## INTRODUCTION

Non-communicable diseases, as chronic diseases, account for 70% of the mortality rates worldwide, while communicable diseases cause the remaining 30%. Among non-communicable diseases, those with high mortality rates include cancer, diabetes, cardiovascular disease, and lung disease. Cancer is classified as a fatal disease for patients because it is difficult to cure owing to its rapid growth, and is highly likely to spread throughout the body through blood or lymphatic fluid. In particular, reduction of female physical activity due to various social environments is a potential cause of increased breast cancer incidence. In addition, the most frequent characteristic of breast cancer among women is not only high incidence, but also high efficiency of cancer treatment. Although the effectiveness of anti-cancer drugs is very high for breast cancer patients, a lot of pain, caused by chemotherapy, accompanies it. Therefore, there is an urgent need to improve the survival rate of breast cancer patients, and improve the quality of life during chemotherapy treatment. Recently, various preclinical studies have suggested that exercise attenuates tumor growth and tumorigenesis (Table 1). However, molecular mechanisms by which exercise affects cancer progression are not yet clear. In this review, we aimed to summarize studies on exercise methods that could potentially increase the survival rate of breast cancer patients and suppress cancer progression.

**Table 1.** Changes in blood variables before exercise and during post-exercise period.

Mouse model	Mouse	Induction	Exercise	Protocols	Test	Efficacy & Signal pathways	Ref
Xenograft	NMRI-Foxn1^nu^	MCF-7 cell (ER^+^, PR^+^, HER2^-^) MDA-MB-231 cell (ER^-^, PR^-^, HER2^-^)	Running	Voluntary wheel running (4 km per night/cage)	-Tumor growth - To evaluate the effect of exercise-conditioned serum in cancer cell	- MCF-7 (–36%, P <0.05) and MDA-MB-231 (–66%, P < 0.01) tumor growth - Regulating Hippo signaling (ANKRD1 and CTGF)	4
Orthotopic	FVB/NJ	p53/PTEN double-null (−/−) primary cell	Stretching	Treated for 10 minutes once a day, for four weeks	Tumor growth	52% reduced tumor size	5
Xenograft	Female BALB/c	MC4-L2 cell (ER^+^, PR^+^)	Running	Using the treadmill; After acclimation, the interval exercise training protocol commenced at 16–18 m min−1, 0% gradient, for 10−14 min, 5 days each week for 6 weeks, and the exercise intensity was gradually increased each week	- Tumor volume - mRNA expression & Protein expression	- Decrease tumor volume and weight - Reduced PI3K/AKT and ERK activation ; Induced Apoptosis	6
Orthotopic	Female APOE^-^^/^^-^	E0771 cell (ER^+^, PR^+^, HER2^+^)	Running	Voluntary wheel running	- Tumor growth & metastasis - Property of tumor	- Increasing log phase tumor growth and inhibiting metastasis - Reduced tumor hypoxia affect exponential tumor growth in APOE^-^^/^^-^mice	7
Orthotopic	Female FVB/NJ Female BALB/c Female C57/BL6	C3(1)SV40Tag -p-16-lu cell (Claudin-low breast cancer) E0771 cell 4T07 cell (ER^-^, PR^-^, HER2^-^)	Running	Using the treadmill; After acclimation, 5 m/ min for 5 min, 10 m/min. for 5 min, 15 m/min for 5 min., and 20 m/min. for 45 min., which is equivalent to 70% VO2 peak. The exercise intensity was gradually increased each week for 2 weeks	-Tumor growth - Gene expression	- 771 (0.5 folds), C3(1) SV40Tag-p16-lu cell (2 folds), 4T07 cell ( same size) tumor size - Ki67 expressions : E0771 (0.25 folds), C3(1)SV40Tagp16- lu cell (1.26 folds), 4T07 cell (same expression) - Hif1-α expressions :E0771 (-5.0 folds), C3(1)SV40Tagp16- lu cell (11.0 folds), 4T07 cell (same expression)	8
Orthotopic	Female C57/BL6	4T1 cell (ER^-^, PR^-^, HER2^-^) E0771 cell	Running	Wheels (running group) vs. without Wheels (sedentary group)	-Tumor growth, perfusion, hypoxia, and components of the antigenic and apoptotic cascades	-Statistically significantly reduced tumor growth and was associated with a 1.4-fold increase in apoptosis	9
Xenograft	Female BALB/c	4T1 cell	Running	Using the treadmill (18 m/min for 30 min once a day) vs. sedimentary group for 30 days	-Tumor growth -Evaluating immune cell ratio	- Exercise regulates tumor growth through immune cells responses - Exercise with radiotherapy reduces MDSCs accumulation and NK cell activation	10
Orthotopic	Female BALB/c	4T1 cell	Running	Low intensity exercise (6 m/min, 60 min/d) group (LE), Medium intensity exercise (10 m/min, 60 min/ d) group (ME), High intensity exercise (15 m/min, 60 min/d) group (HE) one a day for 20 days	- Tumor growth -Evaluating apoptosis signals	- HE inhibited tumor growth - HE combined with administration of didzein induces apoptosis of breast cancer	11
Orthotopic	Female BALB/c	4T1 cell	Running	voluntary exercise four weeks; 170.45±47.5 km and 17.45±1.8 m.min-1,	-Tumor growth	- Beneficial effects of voluntary exercise on breast cancer progression	12
Orthotopic	BALB/cBy	4T1 cell	Running	Using the wheel running: The running group ran an average daily distance of 4.89 ± 1.73 km over 60 days prior to 4T1 tumor cell injection, and 2.38 ± 1.51 km over 30 days after tumor cell injection	-Tumor growth	-Running longer distances is associated with decreased breast tumor burden in old mice	13
Xenograft	Female BALB/c	MCF-7 cell (ER^-^, PR^-^, HER2^-^)	Running	Using the wheel running: 18 m/min for 30 min for days per 12 weeks	-Gene expression	-Exercise decrease the IL- 6, IL-18, TNF-a, CRP mRNA expression	14
Transgenic mice	FVB/NJ C3(1)/SV40Tag	Genetically predisposed to develop breast cancer	Running	Voluntary wheel running : 1 h/day, 6 days/week for 20 weeks	-Voluntary physical activity (Running distance/ Speed) Tumor size	-C2(1)/SV40Tag mice < FVB/ N mice C2(1)/SV40Tag mice > C2(1)/ SV40Tag + exercise	15
Transgenic mice	p53-deficient (p53+/−): MMTV-Wnt-1	Genetically predisposed to develop breast cancer	Running	1) voluntary wheel running Con-WHL, WHL (exercise) 2) non-Voluntary wheel running : Untreated group, 20 m/min (TREX1), 24 m/min (TREX2) for 5 days / weeks	-p53 expression -Incidence -Multiplicity & survival	-Con = TREX1 =TREX2 / Con -WHL = WHL -Con<TREX1=TREX2 / Con- WHL < WHL -Con>TREX1=TREX2 / Con- WHL < WHL	16
Orthotopic	Athymic	MDA-MB-231 cell	Running	Voluntary wheel running running distance range ~4 to ~6 km/day for 15 weeks	- Survival -VEGF -HIF-1alpha expression -tumor metabolism	- Con = Exercise - VEGF expression: Con (48.6 pg/ml > Exercise (47.0 pg/ml) - HIF-alpha expression: Con (5.4.%l > Exercise (11.4%) Con (0.0.34 mmol/g) < Exercise (0.42 mmol/g)	17
Transgenic mice	MMTV-PyMT Tg	Genetically predisposed to develop breast cancer	Running	Voluntary wheel running	-Tumor growth -Heart mass / Spleen mass -cytokine expression	-Con > Exercise -Con < Exercise -CCL22 : Con> Exercise CXCR4: Con <Exercise	18
Orthotopic	Female BALB/c	4T1 cell	Running	Treadmill running progressive time (10-15 min) and Speed (8-12 m/min) for 8 weeks	-Carbohydrate oxidation -Gene expression	-Decrease the carbohydrate oxidation in Exercise group -Up-regulated Ldha, HKII, glut 1, HIF-1a, Mtor, p53, Lats2 expression	19
Xenograft	Female BALB/c	MC4-L2 cell (ER+)	Running	6-18 m/min for 20-30 min for 4 weeks	-Gene expression	The lowest level of IL-6, VEGF	20
Xenograft	Female BALB/c	4T1 cell	Running	Endurance-trained for 8 weeks; mice exercised 5 days a week, for 8 consecutive weeks (In the 8th and final week the mice ran for 26 min a day, spending 1 min at 6 m/min, 1 min at 8 m/min, 22 min at 10 m/min, and 2 min 12 m/min.)	-Tumor growth -Gene expression	-Exercise has -17% growth rate, 24% long survival - 2- folds CD8+/FoxP3+ (Endurance exercise enhances antitumor immune efficacy)	21
Xenograft	Female BALB/c	4T1 cell	Running	Using the treadmill; After acclimation, 5 m/ min for 5 min., 10 m/ min. for 5 min., 15 m/ min. for 5 min., and 20 m/min. for 45 min., which is equivalent to 70% VO2 peak . The exercise intensity was gradually increased each week for 2 weeks	-Gene expression	-Anti-inflammation : IL-10/ TNF-α ratio and IL-15 expression	22
Xenograft	Female BALB/c	4T1 cell	Swimming	Swim training 5 days/ week for 4 weeks	-Gene expression	-Th1 systemic response ; -Gata3 and Foxp3	23
Xenograft	Female BALB/c	4T1 cell	Running	4 weeks of high-intensity interval training (HIIT) and saffron aqueous extract (SAE) supplementation	-Tumor growth -Gene expression	HIIT is associated with a reduced risk of cancer-related muscle wasting; SAE enhances the improvement of muscle loss and apoptotic indices	24
Xenograft	Female Balb/c	MC4-L2 cell	Running	Treadmill 16–18 m/min, 0% grade, 10–14 min, 5 days/week for 5 weeks	-Gene expression	-miR-21 pathways; reduced IL-6 levels, NF-kB and STAT3 expressions & up-regulated TPM1 and PDCD4 expressions	25
Xenograft	Female C57BL/6	EO771 breast tumor cell	Running	Reached maximum ethical size in wheel running (8 km per day)	-Tumor hyposia, perfusion, vascularity and proliferation	unknown	26
Xenograft	Athymic	MDA-MB231 cell	Running	Voluntary exercise; The five-week period ranged from < 1 to 7.9 miles/day	-Tumor growth	-Inhibiting the growth of carcinomas	27
Chemical induced mouse model	Female Balb/c	7,12-dimethylbenzanthracene (1 mg/ml weekly for 6 weeks)	Swimming	physical training of swimming in water (30 ± 4°C) for 45 min (5 times per week for 8 weeks)	-Gene expression	-Reduced Th1 cytokine increasing the Th2 cytokines and Treg cells	28

ER: estrogen receptor, PR: progesterone receptor, HER2: receptor tyrosine-protein kinase erbB-2, PTEN: Phosphatase and tensin homolog, APOE: apolipoprotein E, FVB: Friend leukemia virus B, ANKRD1: Ankyrin repeat domain protein, CTGF: connective tissue growth factor, PI3K: phosphoinosidied 3-kinase, AKT: protein kinase B, ERK: extracellular signal regulated kinase, IL: interleukin, TNF-α: tumor necrosis factor – α, CRP : C-eactive protein, VEGF: vascular endothelial growth factor, HIF-1 α: hypoxia-inducible factor 1- α, CCL2: C-C motif chemokine ligand 2, CXCR4: C-XC chemokine receptor type 4, Ldha: lacate dehydrogenase A, HKII: hexokinase II, Glut 1: glucose transporter 1, Mtor : mammalian target of rapamycin, Lats2: large tumor suppressor kinase 2, CD8: cluster of differentiation 8, FoxP3: forkhead box P3, Gata3: GATA binding protein 3, Th : T helper cell, TPM1: tropomyosin alpha-1chain, PDCD4: programmed cell death protein

### Conventional breast cancer therapy

Modern people often suffer from various diseases, which leads to death. In particular, the four major chronic diseases leading to death have been reported as cancer, diabetes, cardiovascular, and chronic lung diseases. According to the Cancer Society report, the most common cancer among women worldwide is breast cancer^1^. Furthermore, the most common types of cancer in Korean women were breast cancer (19.9%), thyroid cancer (18.8%), colorectal cancer (10.5%), gastric cancer (9.2%), lung cancer (7.3%), and stomach and liver cancers (3.7%) were investigated according to a survey posted on the National Cancer Information Center (NCIC)^2^.

Standard treatment methods such as various anti-cancer drugs and surgery are being developed, and alternative medical technologies for incurable diseases are also in development. Currently, there are four main ways of cancer treatments: 1) surgery, 2) chemotherapy, 3) radiation therapy, and 4) hormone therapy.

Prophylactic surgery suppresses the cancer progression by performing a biopsy for the purpose of diagnosis through surgery or removing the benign tumor completely. Surgery also prevents the spread of cancer to other cells in the body and helps relieve symptoms. Chemotherapy refers to the use of therapeutic agents for regulating hyperproliferative cells. Radiation therapy kills cancer cells by directly irradiating them. Hormone therapy that suppresses estrogen action is also used as a cancer treatment method, as breast cancer is affected by estrogen levels, unlike other cancers.

### Exercise regulates the breast cancer in animal models by inhibiting carcinogenesis

Disease increase over the last two decades may be due to a more westernized lifestyle, which is accompanied by excessive nutrition and lack of exercise^3^. Guidelines on cancer prevention are well known, and include recommendations for controlling metabolism, such as a balanced nutrient intake, eating vegetables, regulating vitamin intake, and controlling weight. Furthermore, exercise can prevent and treat various diseases, and in recent years, research on anti-cancer efficacy has been actively conducted.

Physical activities of Korean women are very low compared to women in other countries. Moreover, many women have adopted western food and a sedentary lifestyle, which has led to reduced voluntary exercise. The highest incidence of cancer among Korean women is breast cancer, and it has been suggested that breast cancer may be related to metabolic problems. Therefore, the effectiveness of exercise for the treatment or prevention of breast cancer should be investigated in future clinical studies.

**Figure 1.**
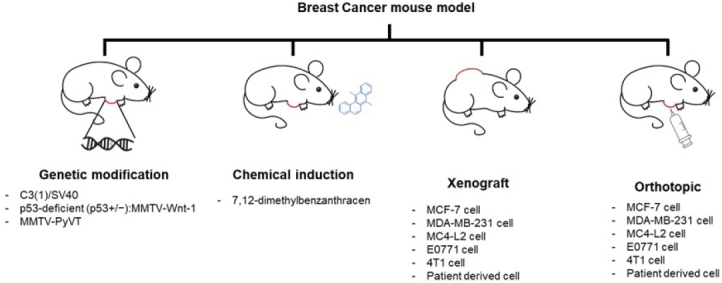
Breast cancer mouse models

An experimental laboratory animal is defined as an animal developed and improved for use in accordance with the purpose of test, diagnosis, education, research, and biological products in the research process. Among laboratory animals, primates such as *Callithrix jacchus* and *Macaca fascicularis* are most similar to humans; however, there exist issues regarding the ethics of conducting research using these animals. Rodents such as *Mus muculus*, *Rattus norvegicus*, and *Cavia porcellus* are the most commonly used experimental animals. In particular, *Mus muculus* has a genetic similarity with humans (approximately >80%), and a biologically similar body structure, a short pregnancy period (19 - 21 days) is also advantageous for preclinical studies. Therefore, *Mus muculus* has been used as a knockout mouse, cancer model xenograft, orthotropic model, and chemically induced-disease model. To develop a mouse model of breast cancer, it is necessary to have experimental cells, and patient derived primary cells such as MCF-7 (ER+, PR+, HER2-), MDA-MD-231 (ER+, PR+, HER2-), MC4-L2 (ER+), E0771 (ER+) and 4T1 (ER-) (Fig. 1).

The breast cancer animal model consists of chemically induced models, transgenic mice models, orthotopic mice models, and xenograft models. In the case of chemically induced breast cancer models, 7,12-dimethylbenzanthracene (DMBA; 1 mg/mL weekly, for six weeks) is injected subcutaneously into the side of the abdomen. Poly-aromatic structure of lipophilic molecule, DMBA has high carcinogen activity in the breast. To evaluate tumor progression, mice are established with genetic modifications that target the oncogene, such as simian virus 40 (SV40) T antigens and polymer middle T antigen (PyMT). In the establishment of mice models by injection with breast cancer cells, mice are mainly used in the study of tumor biology and pharmacology, as these models retain the biological properties of cancer. Breast cancer cells are injected into the mammary fat pad of host mice to obtain orthotropic models. In this case, the number of cells used is appropriate (1 x 10^5^ to 1 x 10^6^/mouse), and cancer cells injected into the mouse organs exhibit properties similar to breast cancer generated in the human body over time, and can be correlated to metastatic cancer. To develop a xenograft model, cells (1 x 10^6^ to 1 x 10^7^/mouse) are injected subcutaneously into the dorsal side of the mouse.

**Figure 2.**
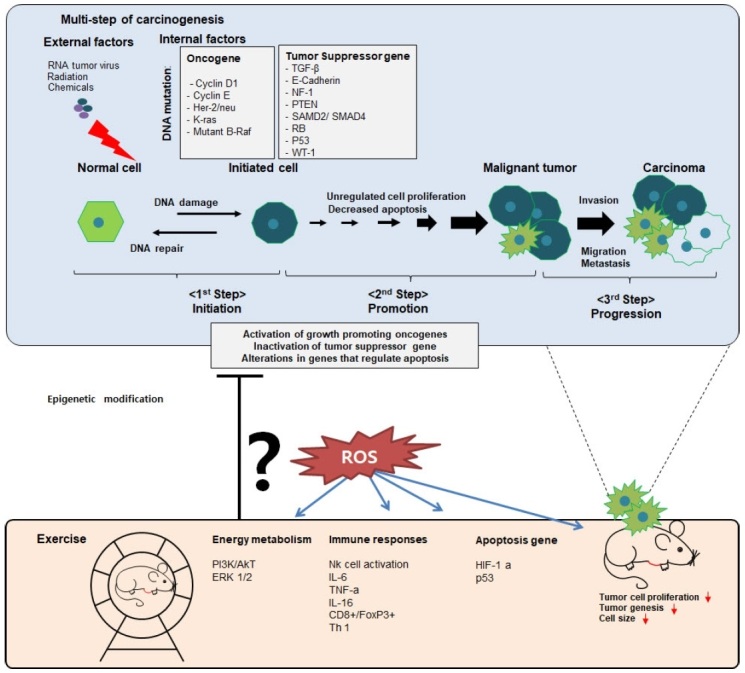
Exercise regulates carcinogenesis by regulating the microenvironment

Using these various animal models, studies on the beneficial effect of exercise against tumor growth and tumorigenesis of breast cancer have been extensively reported (Fig. 2; Table 1). Tumors are defined as transformed cells that undergo abnormal or rapid proliferation, beyond normal regulatory functions, in the organism. Tumors are divided into two types: benign neoplasms and carcinomas. Benign neoplasms have a relatively slow growth rate, and do not penetrate or spread into other tissues. In contrast, carcinomas rapidly grow, and invade other tissues and metastasize. The process of tumor development by carcinogenesis is a multi-step process. The first stage is initiation, where normal cellular DNA is attacked by carcinogens, leading to genetic modification and irreversible mutations. The second stage is promotion, wherein cell proliferation is actively performed to maintain and promote the population of mutant cells to counter immune response in vivo as it eliminates abnormal cells. The third step is progression, the process of increasing the characteristics of a malignant tumor by converting it from a benign tumor to a malignant tumor. In the process of tumor development, the morphology and function of normal cells are altered by genetic modification through internal or external stimulants. External factors include chemical carcinogens such as smoking, physical stimuli such as radiation, and RNA tumor viruses such as HTLV-1 virus. Internal factors involved in the mutation of the target gene include oncogene and tumor suppressor genes. Tumor suppressor genes include TGF-β, E-Cadherin, NF-1, PTEN, SAMD2, SMAD4, and p53, and regulate cell population through apoptosis and proliferation. Oncogene mutation targets are cell cycle regulatory genes such as cyclin D1, Her2, and K-ras. Many preclinical studies suggest that the beneficial effect of exercise training in cancer progression is brought about by direct regulation of intertumoral factors, i.e., tumor growth rate, metastasis, and tumor immunogenicity (Table 1).

Table 1 summarizes the research methods used for controlling the intensity of exercise that underlies the exercise protocols using wheel running, treadmill, and swimming. These preclinical studies clearly demonstrate a decrease in tumor growth rate caused by exercise. Interestingly, Berrueta et al. demonstrated that exercise, such as stretching for 10 minutes once a day over a four-week period, reduced tumor size in a breast cancer model by 50%^5^. In other studies, voluntary exercise also inhibited tumor size and tumor growth ^4,7,9,12,13,15,16-18,27^. Moreover, more studies have been conducted on endurance exercise than resistance exercise; endurance exercise has shown anti-tumor effects^6,8,10,11,21,22^. Taken together, these data suggest that the anti-cancer activity of the exercise protocols is involved in endurance and moderate-intensity exercise.

If so, which mechanism of exercise showed an anti-cancer effect? Results strongly suggest that exercise inhibits epigenetic modification of tumor cells, but enhances apoptosis and immune suppression^29^. Reactive oxygen species (ROS) perform signal transduction in vivo; however, excessive production can cause oxidative stress, which leads to cancer^30^. Moderate intensity exercise can regulate ROS and biological signaling in vivo^31^. It is likely that exercise is related to the regulation of the reactive oxygen species (ROS)-involved microenvironment of cancer^32^. Therefore, these studies also suggest that controlling ROS a potential mechanism for the treatment of cancer^33^.

However, this claim raises further questions as to why exercise is closely related to change in the microenvironment of cancer. One possible belief is that exercise can exert anti-cancer effects by solving problems that arise during metabolic processes. During carcinogenesis, most tumor cells exert cell growth signaling pathway via glucose metabolic reprograming^34^. Recent study suggests that effective anti-cancer effect could be related to the regulation of metabolic syndrome^35^. The results supporting these claims are as follows: First, exercise can lead to activation of natural killer cell, lymphocyte, consequently resulting in the regulation of the tumor growth and metastasis^36^. In addition, exercise attenuates tumorigenesis and tumor progression^37^. Next, the ketone diet (KD) is characterized by high fat, adequate protein, and very low carbohydrate compositions. Some studies have reported that the physiological phenomena caused by exercise or fasting are very similar to physiological conditions observed in the KD^38^. Various preclinical studies have shown that exercise or the KD displays anti-cancer efficacy^39,40^. Taken together, a possible hypothesis is that exercise-binding KD modulates metabolic dysfunction and causes internal factors, which involved in the mutation of the target gene such as ROS generation and tumor-suppressor gene mutations, thereby suggesting its potential as a cancer therapeutic.

However, the anti-cancer effects of KD and exercise can be contradictory. Acute exercise did not change tumor formation, but continuous steady aerobic exercise displayed effective anticancer effects. The general view presented in many studies is that exercise exerts an anti-cancer effect by reducing the size of tumors, promoting energy metabolism, and increasing immune activity by constant exercise. Therefore, further studies should investigate that find and apply an appropriate energy source for exercise that show anticancer efficacy.

## CONCLUSION

Various preclinical studies have shown that exercise weakens tumor growth and tumor development. Moreover, these studies suggest that mice bearing breast cancer exhibited anti-cancer effects by increasing immune responses and anti-inflammatory factor levels through acclimation of increased exercise intensity every week. Thus, continuous exercise can have potential medical benefits as a prevention or therapeutic method for breast cancer. To facilitate this research, researchers need to study the etiological mechanisms that rely on clinical features with underlying pathological features of the disease, as well as based on mechanisms not necessarily present in patients. For example, using animal models to discover new treatments for a variety of diseases is an essential element in discovering new therapeutic targets and performing drug testing at the preclinical stage.

